# Bibliographic Analysis of Oral Precancer and Cancer Research Papers from Saudi Arabia 

**DOI:** 10.31557/APJCP.2020.21.1.13

**Published:** 2020

**Authors:** Shankargouda Patil, Sachin C Sarode, Hosam Ali Baeshen, Shilpa Bhandi, A Thirumal Raj, Gargi S Sarode, Sadiq M Sait, Amol R Gadbail, Shailesh Gondivkar

**Affiliations:** 1 *Department of Maxillofacial Surgery and Diagnostic Sciences, Division of Oral Pathology, *; 4 *Department of Restorative Dental Sciences, Division of Operative Dentistry, College of Dentistry, Jazan University, Jazan, *; 3 *Department of Orthodontics, College of Dentistry, King Abdulaziz University, *; 6 *Center for Communications and Information Technology research, King Fahd University of Petroleum and Minerals, Dharan, Saudi Arabia, *; 2 *Department of Oral Pathology and Microbiology, Dr. D.Y. Patil Dental College and Hospital, Dr. D.Y. Patil Vidyapeeth, Sant-Tukaram Nagar, Pimpri, Pune, *; 5 *Department of Oral Pathology and Microbiology, Sri Venkateswara Dental College and Hospital, Chennai, *; 7 *Department of Dentistry, Indira Gandhi Government Medical College and Hospital, *; 8 *Department of Oral Medicine and Radiology, Government Dental College & Hospital, Nagpur, Maharashtra, India. *

**Keywords:** Bibliometric analysis, oral cancer, oral precancer, Saudi Arabia, scopus

## Abstract

**Objective:**

Oral cancer and precancers are a major public health challenge in developing countries. Researchers in Saudi Arabia have constantly been directing their efforts on oral cancer research and have their results published. Systematic analysis of such papers is the need of the hour as it will not only acknowledge the current status but will also help in framing future policies on oral cancer research in Saudi Arabia.

**Method:**

The search string “oral cancer” OR “Oral Squamous Cell Carcinoma” OR “oral premalignant lesion” OR “oral precancer” OR “Oral Potentially malignant disorder” AND AFFIL (Saudi AND Arabia ) was used for retrieval of articles from Scopus database. Various tools available in Scopus database were used for analyzing the bibliometric related parameters.

**Results:**

The search revealed a total of 663 publications based on the above query. Maximum affiliations were from King Saud University (163) followed by Jazan University (109) and then King Abdulaziz University (106). A large number of international collaborations were observed, the maximum with India (176) and the USA (127). The maximum number of articles were published in the Asia Pacific Journal of Cancer Prevention (34) followed by the Journal of Contemporary Dental Practice (33) and Journal of Oral Pathology and Medicine (19).

**Conclusion:**

Saudi researchers are directing their efforts towards the public health menace of oral cancer. However, it was also observed that some institutions have emerged as front runners in research, whereas others are contributing significantly less. The health department should encourage and take necessary steps to increase the involvement of other institutions.

## Introduction

Oral cancer is reported to be the tenth most commonly diagnosed cancer in the world, with an annual incidence of >300,000 cases. Incidence (3.29%) and mortality (5%) of oral cancer are higher in developing countries when compared with developed countries due to the combined effect of increased life expectancy, and the high or increasing levels of prevalence of cancer risk factors especially the use of smokeless tobacco (Basha et al., 2019; Ferlay et al., 2010). In the Kingdom of Saudi Arabia (KSA) oral cancer is the most common malignancy (17.6%) followed by liver (14%) and lymphoma and leukemia (13.5%). (Ferlay et al., 2010; Tandon et al., 1995; Amer et al., 1985) Among all the head and neck cancers detected yearly in KSA, oral cancer accounts for as much as 26 percent, a majority of them in advanced stage receiving palliative treatment (Gupta et al., 2014). As a result, a large number of authors have focused their efforts to cancer research. 

Research work done by an individual or academic institute is published as research/original articles in popular journals. This is an important method of dissemination of knowledge so that the scientific community becomes aware of the latest developments in the scientific world (Foy et al., 2018). Research publications and academic communication in scientific journals is the most reliable gauge to measure research output of any country, or organization, in any specific branch of knowledge. Bibliometric studies have been carried out to assess the research productivity in different fields of knowledge. (Haq and Al Fouzan, 2018) We also believe that such analysis on important topics such as oral cancer and precancer will help in formalizing appropriate guidelines at national level.

The evolution of the electronic age has led to the development of numerous medical databases on the World Wide Web, offering search facilities on a particular subject and the ability to perform citation analysis. The Scopus database was developed by Elsevier, combining the characteristics of both PubMed and Web of Science. Scopus indexes a wide range of journals and both keyword searching and citation analysis are possible. It offers a quick search, a basic search, an author search, an advanced search, and a source search (Falagas et al., 2008; Moho et al., 2007)

Saudi Arabia has been investing a huge amount and doing progressive efforts to improve the quality of higher education and research output during last two decades (Alhaider et al., 2015). Researchers in KSA have been publishing a large number of papers on the subject of oral oncology. Hence, the present study was conducted to analyze the oral precancer and cancer research published from dental schools of KSA (1984-2019) using Scopus database.

## Materials and Methods

A retrospective observational study was conducted in the dental college of Jazan University, KSA. The research documents produced by the researchers of various dental colleges in KSA were browsed using bibliometric indicators from Elsevier’s Scopus database. Scopus is an abstract and citation database launched by Elsevier in 2004. It covers nearly 40,503 titles from approximately 12,000 publishers, of which approximately 34,000 are peer-reviewed journals in varied fields: social sciences, life sciences, physical sciences, and health sciences. Scopus’ ‘Source Browse’ and ‘Source List’ are refreshed and updated three times per year. To ensure high-quality standards, the journals covered in the Scopus database are reviewed each year. For the present study, the search string “oral cancer” OR “ Oral squamous cell carcinoma” OR “oral premalignant lesion” OR “oral precancer” OR “Oral Potentially malignant disorder” AND AFFIL (Saudi AND Arabia ) was used for retrieval of articles. Boolean operators were used as the present systematic analysis of bibliometrics was a subject related inquiry. ‘Affiliation match’ option was explored to find out any possibility of multiple affiliations of a single university. The data obtained were sorted by year of publication, document type, journal name, author name, affiliation, source of funding, collaboration with other countries and universities, and subject. Various data analysis tools available in Scopus database were used for analyzing the various parameters.

**Figure 1:**
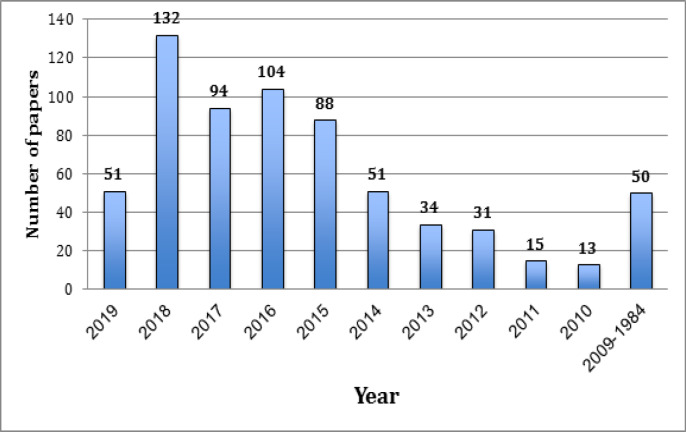
Year-Wise Distribution of the Number of Publications on Oral Precancer and Cancer from Saudi Arabia

**Table 1:** Distribution of the Search Results Based on the Document Type for Oral Precancer and Cancer Research Papers from Saudi Arabia

Sr. No	Document Type	Number	Percentage
1	Article	480	72.40
2	Review	124	18.70
3	Editorial	21	3.17
4	Book Chapter	16	2.41
5	Letter	9	1.36
6	Article in Press	6	0.90
7	Conference Paper	3	0.45
8	Short Survey	2	0.30
9	Note	1	0.15
10	Retracted	1	0.15

**Table 2:** Number of Publication on Oral Precancer and Cancer Research Papers from Saudi Arabia Based on the Journal Title

Source Title	No.
Asian Pacific Journal of Cancer Prevention	34
Journal of Contemporary Dental Practice	33
Journal of Oral Pathology and Medicine	19
Oral Oncology, PLOS One, Saudi Medical Journal	13 each
Journal of International Oral Health, Saudi Dental Journal	7 each
Annals of Saudi Medicine, International Journal of Molecular Sciences, Journal of Cancer Education, Scientific Reports, World Journal of Dentistry, Oral Surgery Oral Medicine Oral Pathology and Oral Radiology	6 each
Current Drug Metabolism, European Journal of Dentistry, Head and Neck, Journal of Periodontology, Oncotarget	5 each
Biomedicine and Pharmacotherapy, Journal of Cancer Research and Therapeutics, Journal of Clinical and Experimental Dentistry, Journal of Oral and Maxillofacial Pathology, Oral Diseases	4 each
Biomedical Research India, Biosciences Biotechnology Research Asia, Clinical Oral Investigations, Community Dentistry And Oral Epidemiology, Eastern Mediterranean Health Journal, Indian Journal Of Dental Research, Inhalation Toxicology, International Dental Journal, International Journal Of Nanomedicine, International Journal Of Pharmacology, International Medical Journal, Journal Of Investigative And Clinical Dentistry, Life Science Journal, Endodontology, Saudi Pharmaceutical Journal, Springer briefs In Public Health, Tropical Journal Of Pharmaceutical Research, Tumor Biology	3 each
Applied Spectroscopy Reviews, Archives Of Oral Biology, Artificial Cells Nanomedicine And Biotechnology, Bioinorganic Chemistry And Applications, Biomed Research International, Biomolecules, British Journal Of Cancer, British Journal Of Dermatology, British Journal Of Oral And Maxillofacial Surgery, Cancer Cell International, Cancers, Carcinogenesis, Clinical And Experimental Dental Research, Current Pharmaceutical Design, Disease A Month, Drug And Chemical Toxicology, Endocrine Related Cancer, European Journal Of Oral Sciences, Evidence Based Complementary And Alternative Medicine, Future Oncology, Genes, Genetic Testing And Molecular Biomarkers, Implant Dentistry, International Journal Of Biological Macromolecules, International Journal Of Cancer, International Journal Of Dentistry, International Journal Of Health Care Quality Assurance, International Journal Of Oncology, International Journal Of Oral And Maxillofacial Surgery, Journal Of Clinical And Diagnostic Research, Journal Of Experimental Therapeutics And Oncology, Journal Of Inorganic And Organometallic Polymers And Materials, Journal Of International Society Of Preventive And Community Dentistry, Journal Of Medical Systems, Journal Of Molecular Liquids, Journal Of Oral And Maxillofacial Surgery, Journal Of Oral And Maxillofacial Surgery Medicine And Pathology, Journal Of Oral Biology And Craniofacial Research, Journal Of Oral Microbiology, Journal Of Physical Chemistry, Journal Of The College Of Physicians And Surgeons Pakistan, Journal Of The Pakistan Medical Association, Laser Physics, Lipids In Health And Disease, Materials, Medicinal Chemistry, Meta Gene, Molecular Carcinogenesis, Molecules, Nigerian Journal Of Clinical Practice, OMICS A Journal Of Integrative Biology, Oncology Letters, Open Dentistry Journal, Pakistan Journal Of Medical Sciences, Photodiagnosis And Photodynamic Therapy, Recent Patents On Anti-Cancer Drug Discovery, Recent Patents On Biomarkers, Rsc Advances	2 each
ACS Nano, ACS Omega, APMIS, Academic Emergency Medicine, Acta Dermatovenerologica Alpina Pannonica Et Adriatica, Acta Microbiologica Et Immunologica Hungarica, Acta Odontologica Scandinavica, Acta Pharmaceutica Sciencia, Advanced Functional Materials, Advances In Clinical Chemistry, Advances In Environmental Biology, Advances In Physiology Education, African Health Sciences, African Journal Of Biomedical Research, Ain Shams Engineering Journal, American Journal Of Biochemistry And Biotechnology, American Journal Of Dermatopathology, American Journal Of The Medical Sciences, Analytical Biochemistry, Angewandte Chemie International Edition, Annals Of Oncology, Anticancer Research, Apoptosis, Applied Ergonomics, Applied Organometallic Chemistry, Arabian Journal For Science And Engineering, Australian Journal Of Chemistry, BMC Cancer, BMC Complementary And Alternative Medicine, BMC Medical Genetics, BMC Oral Health, BMC Public Health, BMC Research Notes, Biochimica Et Biophysica Acta Reviews On Cancer, Bioinformatics, Biomacromolecules, Biomaterials, Biosensors And Bioelectronics, Biotechnology Journal, Bone Marrow Transplantation, Brazilian Journal Of Oral Sciences, Breast Cancer Targets And Therapy, British Journal Of Ophthalmology, Canadian Journal Of Gastroenterology, Cancer, Cancer And Metastasis Reviews, Cancer Biomarkers, Cancer Investigation, Cancer Letters, Cancer Research, Cancer Science, Cell Metabolism, Cell Proliferation, Cellular And Molecular Biology	1 each

**Table 3:** Most Productive Authors for Oral Precancer and Cancer Research Papers from Saudi Arabia

S.no.	Author Name	Affiliation	Number	Percentage
1	Patil, S.	Department of Maxillofacial Surgery and Diagnostic Sciences, Division of Oral Pathology, College of Dentistry, Jazan University, Jazan, Saudi Arabia	69	10.41
2	Sarode, S.C.	Department of Oral Pathology and Microbiology, Dr. D. Y. Patil Dental College and Hospital, Dr. D. Y. Patil Vidyapeeth, Pune, India	43	6.49
3	Sarode, G.S.	Department of Oral Pathology and Microbiology, Dr. D. Y. Patil Dental College and Hospital, Dr. D. Y. Patil Vidyapeeth, Pune, India	39	5.88
4	Javed, F.	Department of Orthodontics, Eastman Institute for Oral Health, University of Rochester, New York, United States	22	3.32
5	Awan, K.H.	College of Dental Medicine, Roseman University of Health Sciences, Utah, South Jordan, United States of America	19	2.87
6	Al-Maweri, S.A.	Department of Oral Medicine and Diagnostic Science, Al Farabi Colleges, Riyadh, Saudi Arabia	14	2.11
7	Tarakji, B.	Department of Oral and Maxillofacial Sciences, AL-Farabi Colleges of Dentistry and Nursing, Riyadh, 11691, Saudi Arabia	13	1.96
8	Warnakulasuriya, S.	Department of Oral Medicine, King’s College London and WHO Collaborating Centre for Oral Cancer, London, United Kingdom	13	1.96
9	Divakar, D.D.	Dental Biomaterials Research Chair, Dental Health Department, College of Applied Medical Sciences, King Saud University, Riyadh, Saudi Arabia	10	1.51
10	Gadbail, A.R.	Government Medical College Nagpur	10	1.51
11	Kamal, M.A.	King Fahd Medical Research Center, King Abdulaziz University, Jeddah, Saudi Arabia	10	1.51
12	Khurshid, Z.	Department of Prosthodontics and Dental Implantology, College of Dentistry, King Faisal University, Al-Hofuf, Saudi Arabia	10	1.51

**Table 4:** Top Ten Institutional Affiliations for Oral Precancer and Cancer Research Papers from Saudi Arabia

S.No.	Affiliation	Number	percentage
1	King Saud University	163	24.58
2	Jazan University	109	16.44
3	King Abdulaziz University	106	15.98
4	Dr. D.Y.Patil Dental College & Hospital	47	7.08
5	King Faisal Specialist Hospital and Research Centre	33	4.97
6	Taibah University	26	3.92
7	King Saud University College of Applied Medical Sciences	26	3.92
8	Imam Abdulrahman Bin Faisal university	25	3.77
9	King Saud University College of Science	23	3.46
10	Umm Al Qura University	23	3.46
11	Al Qassim University	23	3.46
12	King Khalid University	22	3.31

**Table 5:** International Collaborations Associated with Oral Precancer and Cancer Research Papers from Saudi Arabia

Sr. no.	Country/Territory	Number
1	India	176
2	United States	127
3	Egypt	76
4	United Kingdom	66
5	Pakistan	50
6	Australia	38
7	Canada	31
8	Yemen	23
9	China, Germany, Italy, Malaysia	20 each
10	Brazil, Sudan	13 each
11	Japan, South Korea	12 each
12	Singapore	11
13	Finland, Syrian Arab Republic	10 each
14	Jordan	9
15	Iran, United Arab Emirates	8 each
16	France	7
17	Taiwan	6
18	Bangladesh, Libyan Arab Jamahiriya, Netherlands, South Africa, Turkey	5 each
19	Lebanon, Poland, Spain, Sweden	4 each
20	Austria, Bahrain, Greece, Israel, Kuwait, Portugal, Qatar	3 each
21	Belgium, Colombia, Denmark, Estonia, Hong Kong, Kazakhstan, Norway, Oman, Romania, Russian Federation, Vietnam	2 each
22	Cameroon, Hungary, Indonesia, Iraq, Ireland, Mauritius, Monaco, Nigeria, Palestine, Sri Lanka, Switzerland, Tanzania, Tunisia, Venezuela	1 each

**Table 6:** Subject-Wise Distribution of the Publications on Oral Precancer and Cancer from Saudi Arabia

Sr. no.	Subject Area	Number
1	Medicine	337
2	Biochemistry, Genetics and Molecular Biology	240
3	Dentistry	168
4	Pharmacology, Toxicology, and Pharmaceutics	85
5	Chemistry	45
6	Agricultural and Biological Sciences	32
7	Chemical Engineering	30
8	Engineering	30
9	Materials Science	29
10	Environmental Science	19

## Results

The search revealed a total of 663 publications over a period of 35 years. It can be observed that the publication productivity on oral cancer and precancer has seen a remarkable rise in the last decade (Figure 1). Only 7.5% of the total documents were published from the year 1984 to 2009. However, since 2010, the number of publications has increased manifolds. The years 2018 and 2016 were the most productive with 132 and 104 publications respectively. The total number of citations received by the 663 publications were 7403 with h index of 39. 

Based on the document type, it was observed that about 72% (n=480) of the documents were original articles, and approximately 20% (n= 124) were review articles. The remaining were Editorials (21; 3.17%), Book Chapters (16; 2.41%), Letters (9; 1.36%), Article in Press (6; 0.9%), Conference Papers (3; 0.45%), and Short Surveys (2; 0.3%) (Table 1).

The manuscripts were published in 153 different journals. The maximum number of articles (n=34) were published in the Asia Pacific Journal of Cancer Prevention followed by Journal of Contemporary Dental Practice (33), and, Journal Of Oral Pathology And Medicine (19). In addition, there were a large number of journals where only 1-3 articles appeared (Table 2). 

The 663 publications were authored by 160 researchers. Here, the names of the top ten contributors are included (Table 3). These are: Dr. S. Patil has the maximum publications (69) to his credit, followed by Dr. S.C. Sarode (42) and Dr. G.S. Sarode (39). Of the 160, twelve authors have published 10 or more papers on the subject of oral cancer and pre-cancer.

Additionally, the authors were also checked for their institutional affiliations. The top twelve contributing universities/institutions are listed in Table 4. The highest number of authors were affiliated to King Saud University, followed by Jazan University, and King Abdulaziz University. It is interesting to note that more than half the publications were from these twelve institutions.

The publications were also sorted based on international collaboration. Saudi researchers worked together with researchers from 65 other countries. However, the maximum number of collaborations were with Indian authors (176) followed by United States (127) and Egypt (76) (Table 5).

The 663 papers on oral cancer and precancer were published under 25 different departments/subjects. The maximum articles were authored by the Medicine Department followed by Biochemistry, Genetics and Molecular Biology, and, Dentistry (Table 6).

Finally, the source of funding for the various research papers was examined. Of the 663 publications, only 293 papers declared any funds/grants. The highest number of papers were funded by King Saud University (44), followed by King Faisal University (22) and then King Abdulaziz University (15).

## Discussion

The present study aimed to quantify the oral precancer and cancer research published from Saudi Arabia (1984-2019) based on keyword search conducted on the Scopus database. The results of the search were sorted based on year of publication, document type, journal name, author name, affiliation, source of funding, collaboration with other countries and universities, and subject. It was encouraging to note the interest that investigators from KSA have shown in cancer and pre-cancer research.

Oral cancer prevalence in KSA has been reported to range from 21.6% to 68.6%.(Basha et al., 2019; Ferlay et al., 2010) Numerous studies have listed the use of Shamma, a form of smokeless tobacco, to be associated with the aforementioned high incidence of oral cancer. (Amer et al., 1985; Alsanosy, 2014; Brima, 2016) Not unexpectedly, oral cancer research is being extensively encouraged in all institutions in KSA. 

Scopus is the largest abstract and citation database of peer-reviewed literature: scientific journals, books, and conference proceedings. All journals covered in the Scopus database are reviewed each year to ensure that high-quality standards are maintained. Scopus also offers author profiles which cover affiliations, several publications and their bibliographic data, references, and details on the number of citations each published document has received (Meho et al., 2007; Falagas et al., 2008). Hence, in the present study, the Scopus database was used for retrieval of information.

The extensive literature search revealed that no previous studies have been carried out to evaluate the growth of research on oral cancer and pre-cancer in KSA. It can be observed from the present study that the number of publications from KSA on the subject of oral cancer and pre-cancer has multiplied substantially over the past decade. A similar study was conducted by Ikram Ul Haq et al., (2017) to evaluate the growth of publications on oncology produced by King Saud bin Abdulaziz University for Health Sciences up to December 2015. They reported that there are 45 articles written on the subject of oncology in 19 different journals from KSAUHS. Another study compared the citation performance of cancer research in four Nordic countries: Denmark, Finland, Norway, and Sweden. The study covered articles, obtained through Medline, published during 1978-82. Sweden contributed most cancer articles followed by Denmark. Luukkonen-Gronow, (1988) Another study was conducted by Foy et al., (2018) to provide a descriptive overview of the global research activity on Oral Erythroplakia and Leukoplakia over the past decades. A total of 5,098 published items (articles or reviews) were identified. Also, the USA, India and the United Kingdom were identified as the most prominent countries and contributed to 22%, 11% and 8% of publications respectively.

In the present analysis, Asian Pacific Journal of Cancer Prevention tops among the other journals with 34 research papers. The journal was launched in 2000 as the official publication of the Asia Pacific Organization for Cancer Prevention. Journal provides a forum for communication and propagation of original and innovative research findings that have relevance to understanding the etiology, progression, treatment, and survival of patients. The journal is known for the highest standards of research communication within the cancer sciences community across Asia as well as globally.

Impact factor is very important measure that rank the journal based on the number of citations and usually it designate the reputation of the journal. Among the journals targeted for publishing papers, some of the articles have published in best five impact factor journals such as Cancer Metabolism (IF: 20), Annals of Oncology (IF: 13.92), Biosensors and Bioelectrons (IF: 9.5), Cancer Research (IF: 9.13) and Cancer Letters (IF: 6.4). Other important journals are Cancer (IF: 6.1), British Journal of Cancer (IF: 5.9), Biomicromolecules (IF: 5.6), Biomolecules (IF: 4.6) and Bioinformatics (IF: 4.5).

Alhaider et al. (2015) measured the pharmaceutical research publication from KSA during 2001-2010. The international collaboration share was 40.55%. They reported that King Saud University was the most productive organization with 690 publications (49.75%). This finding is similar to that of the present study where we observed that the maximum affiliations were from King Saud University. Shehata and Mahmood (2016) conducted a bibliometric analysis of publications produced by the researchers of KSA during 1980-2014 with data from Thomson Reuters Web of Science. King Saud University reported 30.85% of the publications.

In the present study, less than half the papers declared any source of funding for research. As lack of grants is a major deterrent to good quality research, this is an area of concern and should be further investigated (Hegde et al., 2017) The researchers should be made aware of the various agencies that are available to fund oral cancer research.

Researchers turn to citation tracking to find the most influential articles for a particular topic and to see how often their own published papers are cited. Citations are assumed to gauge the actual recognition of a piece of work and its tangible impact at the international research front in the short term (Luukkonen-Gronow, 1988). KSA has been at the forefront of biomedical research in the past decade. The authorities have been directing resources and funds towards research in universities. It is encouraging to note the increase in the number of publications, collaborations, and the reach of Saudi research globally (Haq et al., 2018; Latif, 2015; Tadmouri et al., 2019).

The present paper was utilizied only the Scopus database, but future researchers may include the bibliographic citations from Google Scholar and Web of Science databases. Also, the present research was quantitative. A more comprehensive qualitative evaluation of the papers published on specific subjects should be conducted. The papers published in the journals that were not indexed in Scopus were automatically excluded from the present analysis. It is quite possible that some impactful research could be published in such non-indexed journals that could change the results of present analysis. However, based on the reputation and scientific community acceptance, non-inclusion of such papers will not dilute the scientific value of the present paper. 

In conclusion, as reflected in the present paper, Saudi researchers showed significant awareness about oral cancer research. Institutions such as King Saud University, Jazan University and King Abdulaziz University are front-runners in oral cancer and pre-cancer research. Other university’s contributions are comparatively very less. The health department should encourage and take necessary steps to increase the involvement of other institutions. This can be done by creating special funding schemes for the research on oral cancer and precancer and encouraging inter-institutional collaborations with the front-runner institutions. Use of single database and lack of quality analysis of the papers are the limitations of the present study. Future studies should focus on comparative analysis among different databases and identification of the level of evidences using proper scales. We also recommend similar analysis across the globe so that one could understand the top knowledge producing countries in the field of oral precancer and cancer. 
